# Vitamin C Inhibits Scale Drop Disease Virus Infectivity by Targeting Nrf2 to Reduce Ferroptosis

**DOI:** 10.3390/antiox14050576

**Published:** 2025-05-10

**Authors:** Jiaming Chen, Yuting Fu, Shaoping Weng, Jianguo He, Chuanfu Dong

**Affiliations:** 1 State Key Laboratory of Biocontrol, School of Life Sciences, Sun Yat-sen University, Guangzhou 510275, China; chenjm278@mail2.sysu.edu.cn (J.C.); fuyt23@mail.sysu.edu.cn (Y.F.); lsswsp@mail.sysu.edu.cn (S.W.); lsshjg@mail.sysu.edu.cn (J.H.); 2 Institute of Aquatic Economic Animals, Guangdong Provincial Key Laboratory of Aquatic Economic Animals, Sun Yat-sen University, Guangzhou 510275, China; 3 Southern Marine Science and Engineering Guangdong Laboratory (Zhuhai), Zhuhai 519000, China

**Keywords:** scale drop disease virus, ferroptosis, antioxidant, vitamin C, Nrf2, inflammation

## Abstract

Scale drop disease virus (SDDV) poses an escalating threat to global aquaculture, prompting an urgent need for research. Our study found that SDDV infection upregulates genes related to iron, oxidative stress, and lipid metabolism, causing iron overload, reactive oxygen species (ROS) accumulation, and ultimately ferroptosis. Among the tested antioxidants, vitamin C (VC) demonstrated the most potent inhibitory effect in mandarin fish, reducing SDDV-induced mortality by 37.5%. qPCR and IFA results showed that VC effectively suppressed SDDV infection; decreased ROS, lipid peroxidation (LPO), and iron levels; and enhanced glutathione peroxidase 4 (GPX4) expression in infected cells. Mechanistically, VC’s inhibitory effect was reversed by the nuclear factor erythroid 2-related factor 2 (Nrf2) inhibitor ML-385, indicating an Nrf2-dependent pathway. VC promoted Nrf2 nuclear translocation and activated downstream antioxidant genes. Moreover, VC modulated inflammation by regulating pro- and anti-inflammatory factors. These findings suggest VC as a promising therapeutic for SDDV infection.

## 1. Introduction

Scale drop disease virus (SDDV) is a newly identified double-stranded DNA virus with a linear genome that is classified under the *Megalocytivirus* genus in the *Iridoviridae* family [1]. SDS-related cases were first reported in the early 1990s in Asian seabass *Lates calcarifer* farms in Penang, Malaysia [2]. Since then, SDDV has spread widely across Southeast Asia, including to Singapore, Malaysia, Thailand, and Indonesia, becoming a major threat to the bass aquaculture industry in these regions [1,2,3,4]. The cumulative mortality in SDDV-infected Asian seabass can reach up to 40–50%, significantly impacting the economic sustainability of aquaculture [1,2,3,4]. Beyond its established impact on Asian seabass, SDDV has also been reported in a growing number of fish species worldwide, including European chub (*Squalius cephalus*) in England [5], yellowfin seabream (*Acanthopagrus latus*) in China [6], and tilapia (*Oreochromis* spp.) in the USA [7]. Additionally, even at low doses, SDDV has been found to induce complete mortality (100%) in mandarin fish (*Siniperca chuatsi*), suggesting it as a potential host for SDDV transmission and thus its utility as an animal model for studying the infection mechanism of SDDV [8]. These findings underscore SDDV’s potential as a significant global threat to bony fish aquaculture. Therefore, developing effective strategies to control or prevent SDDV infection is crucial for safeguarding global aquaculture industries and ensuring sustainable fish farming practices.

Ferroptosis, a form of regulated cell death characterized by the accumulation of iron and reactive oxygen species (ROS), has been identified as the primary mechanism by which SDDV induces cell death during infection [9]. SDDV infection promotes iron accumulation by targeting TfR1, leading to excessive lipid peroxide buildup that induces ferroptosis [9]. The nuclear factor erythroid 2-related factor 2 (Nrf2) functions as an essential transcription factor that plays a pivotal role in the regulation of iron accumulation and lipid peroxidation (LPO) [10]. Under oxidative stress conditions, Nrf2 translocates to the nucleus, where it activates the expression of genes involved in antioxidant defense, thereby protecting cells from oxidative damage [11]. Evidence suggests that activation of the Nrf2 signaling pathway provides a protective effect against ferroptosis, a process associated with the development of several retinal degenerative disorders [12,13]. Antioxidants such as melatonin and thymoquinone can stabilize redox homeostasis by upregulating Nrf2 and glutathione peroxidase 4 (GPX4) expression, thereby reducing LPO and ROS levels and inhibiting ferroptosis [14]. Thus, Nrf2 serves as a pivotal component in the cellular defense against ferroptosis.

Vitamin C (VC), given its essential role in cellular redox regulation, has emerged as a promising antioxidant capable of mitigating oxidative stress by neutralizing ROS and reactive nitrogen species [15]. As a potent reducing agent, VC stabilizes redox homeostasis by donating electrons to free radicals, thus forming the ascorbate radical, which is subsequently converted to dehydroascorbic acid. It also aids in regenerating other antioxidants such as vitamin E and BH4 [16]. Although the antioxidant properties of VC are well established, its potential role in modulating ferroptosis has not yet been fully explored [17]. Studies have shown that ascorbic acid at concentrations of 0.1–0.5 mM can inhibit ferroptosis [18] and that intraperitoneal administration of VC (150 μL, 1.5 M) reduces ferroptosis in hepatocytes by upregulating SLC7A11/GPX4 expression in mice [19]. Moreover, VC has been shown to exert anti-inflammatory effects by regulating inflammatory cytokines and suppressing immune cell infiltration, both in animal models and clinical studies [20,21,22,23,24,25,26]. In the context of ferroptosis, which can trigger immune responses and inflammation [27], VC’s antioxidant and anti-inflammatory properties make it a theoretically effective candidate for mitigating the ferroptosis and inflammation associated with SDDV infection.

## 2. Materials and Methods

### 2.1. Cell Line and Viral Strain

The cell line mandarin fish fry (MFF-1) was developed and characterized in our laboratory [28]. The cells were grown in Dulbecco’s modified Eagle’s medium (DMEM) enriched with 10% fetal bovine serum (FBS) (Invitrogen, Carlsbad, CA, USA) and kept at 27 °C in a 5% CO_2_ environment [28]. The SDDV strain ZH-06/20 was sourced from the ascitic fluid of yellowfin seabream infected with SDDV in 2020 [6]. The virus was propagated in MFF-1 cells and stored at −80 °C until use.

### 2.2. Artificial Infection

Mandarin fish (50 g) were obtained from Nanhai District, Guangdong Province, China. During the experiment, the fish were randomly tested and found to be free from SDDV and other common viruses, as previously described [6], and assigned to different experimental groups. This protocol was approved by Sun Yat-sen University (Approval No. SYSU-LS-IACUC-2024-0031). 

### 2.3. VC Treatment and Grouping Strategy in MFF-1

For the VC treatment of MFF-1 cells, they were cultured in 24-well plates and incubated with varying concentrations of VC (Beyotime, Shanghai, China), which was diluted in PBS, at 27 °C for 4 h. The control group was incubated with PBS alone. The toxic effect of different VC concentrations on the cells was assessed using the CCK-8 assay. This was followed by infection with ZH-06/20 (MOI = 5) for 1 h. After infection, the cells were washed 3 times with 1 mL of PBS and placed in 0.5 mL of DMEM supplemented with 5% FBS and then incubated at 27 °C in a CO_2_ incubator. The mock group was neither treated with VC nor infected. For antiviral assays, IFA was performed to detect the fluorescence of SDDV major capsid protein (MCP), and qPCR was used to measure the SDDV copy number at 1, 2, and 3 days post infection (dpi). These methods focused on assessing antiviral activity. For ferroptosis assays, cells were cultured in 6-well plates (cultured in 2 mL DMEM after 3 mL PBS washing) and samples were collected at 6, 12, 24, 48, and 72 h post infection (hpi) for subsequent ROS level, LPO content, GPX4 level, and both Fe^2+^ and total iron concentration analyses.

### 2.4. ML-385 Treatment and Grouping Strategy in MFF-1

MFF-1 cells were seeded into 24-well plates and treated with ML-385 (MCE, Monmouth Junction, NJ, USA) at varying doses (3 μM, 5 μM, and 10 μM) for 4 h under 27 °C incubation. Cell viability was determined using the CCK-8 assay. Genes unrelated to the Nrf2 pathway, including *HSP90*, *NF-κB*, and *SLC40A1*, were assessed to verify the specificity of ML-385 via qRT-PCR, while the transcriptional expression of *Nrf2* and its downstream antioxidant genes *HO-1*, *NQO1*, and *GPX4* was quantified via qRT-PCR to verify the effectiveness of ML-385. Additionally, Western blot analysis was employed to measure the protein abundance of Nrf2 and GPX4. Based on the integrated results from viability and molecular assays, 5 μM ML-385 was identified as the optimal concentration for further experimental procedures.

The cells were categorized into 5 experimental groups: DMSO (DMSO diluted with PBS to 20%, ZH-06/20, MOI = 5), ML-385 (5 µM + ZH-06/20, MOI = 5), VC (75 mg/mL VC + ZH-06/20, MOI = 5), positive control (10 µM ferrostatin-1 + ZH-06/20, MOI = 5), and ML-385 + VC (5 µM ML-385 + 75 mg/mL VC + ZH-06/20, MOI = 5). After adding the drugs to the 6-well plates, the cells were incubated at 27 °C for 4 h. Following this, the cells were infected with ZH-06/20 for 1 h, washed 3 times with 3 mL of PBS, and then cultured in 2 mL of 5% DMEM. The SDDV MCP copy number was measured using qPCR at 6, 12, 24, 48, and 72 hpi. ROS and LPO levels were assessed at 1, 2, and 3 dpi.

### 2.5. Transcriptome Sequencing (RNA-Seq)

Spleen tissues from mandarin fish were randomly collected at 5 and 10 dpi, with three fish per group and 500 ng of RNA used for sample preparation from each sample. Sequencing was performed by Shenzhen Gene Genius Biotechnology Co., Ltd. (Shenzhen, China). Processed sequencing reads were mapped to the mandarin fish reference genome (Accession No.: ASM2008) via HISAT2, followed by transcript abundance estimation using featureCounts, version 2.0.1. Differential expression analysis was performed with DESeq2, version 1.47.0, applying statistical thresholds of false discovery rate (FDR) < 0.05 and absolute log2 fold change > 1 to identify significant genes. Functional annotation of differentially expressed genes (DEGs) was carried out through Kyoto Encyclopedia of Genes and Genomes (KEGG) pathway analyses to elucidate their biological roles.

### 2.6. Quantitative Reverse Transcription PCR (qRT-PCR)

The primers used are listed in Table 1. The assay was performed according to established protocols [6]. Briefly, *β-actin* was employed as a housekeeping gene. RNA isolation and subsequent cDNA synthesis were performed using the Evo M-MLV RT Premix for qPCR (Accurate Biology, Changsha, China) according to the manufacturer’s guidelines. Quantitative reverse transcription PCR was executed on a LightCycler^®^ 480-II system (Roche Diagnostics, Indianapolis, IN, USA) with the following reaction components: 10 μL SYBR qPCR Master Mix (Accurate Biology), 2 μL cDNA template, 0.5 μL forward/reverse primers, and 7 μL nuclease-free water. Thermal cycling conditions comprised an initial denaturation at 95 °C for 1 min, followed by 40 cycles of 95 °C for 10 s and 60 °C for 30 s. Melt curve analysis was conducted by heating from 95 °C to 60 °C (1 min) and back to 95 °C, with a final hold at 50 °C for 30 s. Technical triplicates were included for all samples, and relative gene expression was normalized using the 2^−ΔΔCt^ method [29].

### 2.7. SDDV Copy Number Detection

Total genomic DNA was isolated from SDDV-infected MFF-1 cells using a commercial DNA extraction kit (Vazyme, Nanjing, China). Viral copy numbers were quantified using an absolute qPCR assay performed on a Roche LightCycler 480 system, targeting the *MCP* gene of SDDV. Gene-specific primers for *MCP* (forward: 5′-AAG AGC GTG AAG CAA TGT C-3′; reverse: 5′-GGG ATG ACT AAA TCG CAG A-3′) were designed, and a standard curve was generated using serially diluted pMD-19T-MCP plasmid containing the cloned *MCP* sequence. The thermal cycling conditions followed the protocol described in Section 2.6. All DNA samples were analyzed in triplicate, and absolute *MCP* gene expression levels were calculated using a plasmid standard curve as a reference.

### 2.8. Reactive Oxygen Species (ROS) Assay

ROS levels were measured using DCFH-DA (Aladdin Scientific, Shanghai, China), a fluorescent probe that is commonly used to detect reactive oxygen species in cells. Cells were incubated with the probe for 20 min at 27 °C, followed by washing to remove excess probe. Fluorescence intensity was then measured using a Nikon Ti2-E fluorescence microscope (Nikon, Tokyo, Japan).

### 2.9. Lipid Peroxidation (LPO) Assay

LPO levels were assessed using an LPO assay kit (Solarbio, Beijing, China) following the manufacturer’s protocol. Briefly, cells were lysed in 1 mL extraction buffer, followed by centrifugation (8000× *g*, 10 min). Sequential addition of Reagents 1–3 was performed as per protocol specifications. A blank control containing distilled water and a calibration curve derived from serially diluted standards were included. After reagent incubation, samples underwent thermal treatment (100 °C, 60 min) and secondary centrifugation (8000× *g*, 10 min). Dual-wavelength spectrophotometric measurements (532 nm and 600 nm) were acquired, with LPO concentrations calculated by substituting the obtained values into the formula derived from the standard curve.

### 2.10. Cell Viability Assay

Cell viability was assessed using the CCK-8 assay kit (Solarbio, Beijing, China). After the cells were treated with ML-385, 10 µL of CCK-8 solution was added to each well. The cells were then incubated for 1 h, followed by washing with PBS. The absorbance at OD_450_ was measured using a spectrophotometer. The cell viability was calculated by comparing the *OD* values of the experimental group to those of the control group using the following formula: Cell viability (%) = ((OD_treated_ − OD_blank_)/(OD_control_ − OD_blank_)) × 100, where OD_treated_ is the absorbance of the treated group, OD_control_ is the absorbance of the control group, and OD_blank_ is the absorbance of the blank group (without cells).

### 2.11. Western Blot Analysis

Protein samples from MFF-1 cells were extracted using RIPA lysis buffer (Thermo Fisher Scientific, Waltham, MA, USA). Equal protein quantities were separated on 10% SDS-PAGE gels and transferred to PVDF membranes (Merck Millipore, Darmstadt, Germany). Membranes were probed with primary antibodies: GPX4 pAb (HuaBio, Hangzhou, China) or Nrf2 pAb (HuaBio, Hangzhou, China) at a 1:500 dilution. Loading controls included β-actin mAb (Merck Millipore, Darmstadt, Germany) or Lamin b mAb (Proteintech, Chicago, IL, USA) at 1:1000 dilutions. HRP-conjugated goat anti-rabbit/mouse IgG (Invitrogen, Carlsbad, CA, USA) served as the secondary antibody. High-sig ECL substrate was used to detect protein bands (Tanon, Shanghai, China).

### 2.12. Immunofluorescence Assay (IFA)

MFF-1 cells were treated with primary antibodies: MCP mAb at a 1:500 dilution or Nrf2 pAb at a 1:200 dilution. The secondary antibodies used were Alexa Fluor 555-conjugated goat anti-mouse or anti-rabbit IgG (Invitrogen, Carlsbad, CA, USA). Positive controls included VC-treated and SDDV-infected cells, while negative controls were PBS-treated, SDDV-infected cells. After washing with PBS, the cells were stained with Hoechst 33342 (Invitrogen, Carlsbad, CA, USA) to visualize the nuclei, then covered with a mounting medium. The samples were observed and imaged using an Leica TCS SP8 confocal microscope (Leica, Wetzlar, Germany). 

### 2.13. Statistical Analysis

The data are expressed as mean values ± standard deviation (SD) based on three independent experiments. Statistical analysis was carried out using SPSS 16.0. Comparisons between control and experimental groups were conducted using Student’s *t*-test and one-way analysis of variance (ANOVA). Statistical significance was considered when 0.01 < * *p* < 0.05, ** *p* < 0.01, and non-significant differences were marked as “ns”. Groups were labeled with different letters (a, b, c, d) to denote significant differences.

## 3. Results

### 3.1. SDDV-Induced Ferroptosis in the Spleen Tissues of Mandarin Fish

**Figure 1:**
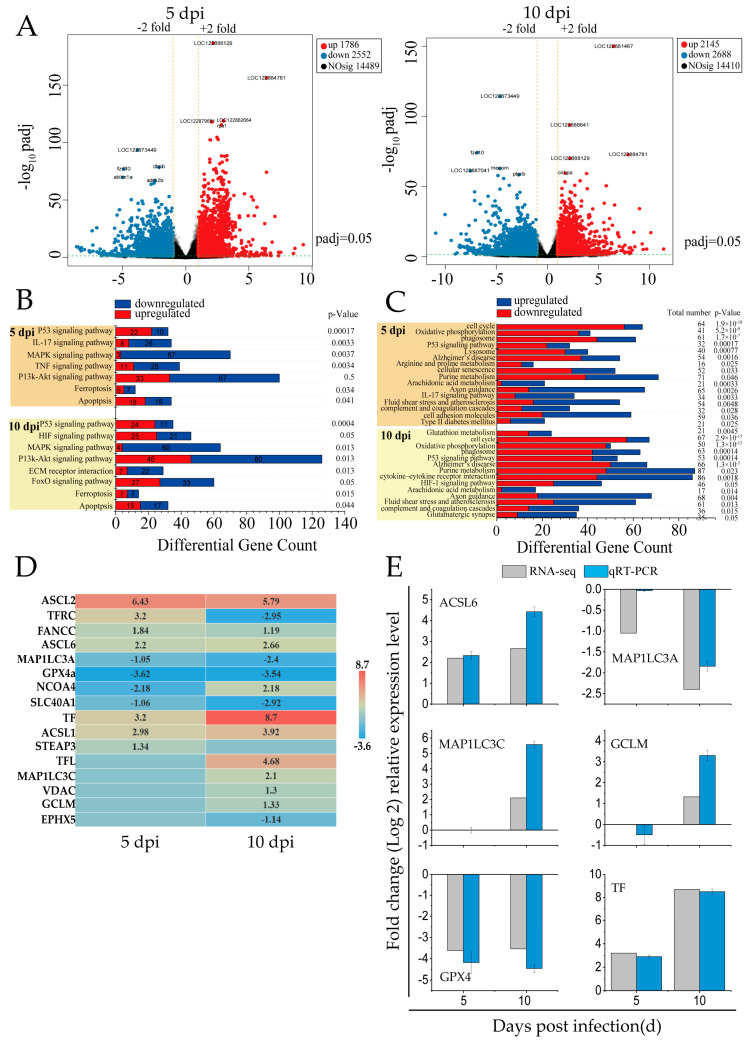
RNA-seq analysis of DEGs related to cell death in the spleens of SDDV-infected mandarin fish. (**A**) Differential gene expression analysis comparing SDDV at 5 and 10 dpi with the control group; (**B**) enrichment of cell-death-related signaling pathways induced by SDDV infection (*p* < 0.05); (**C**) enrichment of ferroptosis-related signaling pathways induced by SDDV infection (*p* < 0.05); (**D**) expression of ferroptosis-related genes induced by SDDV infection (*p* < 0.05); (**E**) validation with qRT-PCR. n = 3.

In this study, RNA-seq analysis was performed on the spleen tissues of mandarin fish infected with SDDV at 5 dpi and 10 dpi. Following SDDV infection at different time points, 14,410 to 14,489 DEGs were identified, including 1786 to 2145 upregulated genes and 2552 to 2688 downregulated genes (Figure 1A).

KEGG pathway enrichment analysis of cell-death-related DEGs revealed that the modes of death included ferroptosis and apoptosis, with ferroptosis being the primary mode induced by SDDV in spleen tissues (Figure 1B). The key regulatory pathways included the P53, IL-17, HIF-1, MAPK, ECM–receptor interaction, PI3K-Akt, TNF, and FOXO signaling pathways.

Ferroptosis-related signaling pathways showed significant alterations at both 5 and 10 dpi (Figure 1C). Oxidative phosphorylation was consistently downregulated, indicating mitochondrial dysfunction and elevated ROS—hallmarks of ferroptosis. Alterations in arachidonic acid metabolism and glutathione metabolism suggested increased lipid peroxidation and compromised antioxidant defenses, contributing to ferroptotic cell death. Furthermore, HIF-1 signaling was upregulated at 10 dpi, potentially promoting iron accumulation and exacerbating cellular damage. Additionally, other pathways such as the P53 signaling pathway and cell cycle regulation were significantly affected, indicating iron metabolism dysregulation and suggesting that cell cycle disruptions may enhance cellular susceptibility to ferroptosis. Upregulation of immune-related pathways, including the complement and coagulation cascades and cytokine–receptor interactions, indicated heightened inflammatory responses during infection.

The infection caused by SDDV resulted in a notable modification in the expression levels of 16 genes associated with ferroptosis (Figure 1D). In iron metabolism, key changes included the downregulation of *TFRC* (*TfR1*) and *SLC40A1* and upregulation of *TF*, *STEAP3*, and *NCOA4*. In glutathione (GSH) metabolism, *GPX4* was downregulated, while *GCLM* was upregulated. The lipid metabolism genes *ACSL1*, *ACSL6*, and *MAP1LC3C* were also upregulated. Additionally, *FANCC* upregulation indicated oxidative stress, as it helps regulate ROS levels to mitigate excessive lipid peroxidation, which is a characteristic feature of ferroptosis. To validate the RNA-seq results, six ferroptosis-related genes were randomly selected for qRT-PCR validation, showing that the results were reliable (Figure 1E).

In summary, SDDV infection induces ferroptosis in the spleen through the modulation of iron metabolism, oxidative stress, and immune responses.

### 3.2. VC Reduces Mortality in Mandarin Fish Infected with SDDV

**Figure 2:**
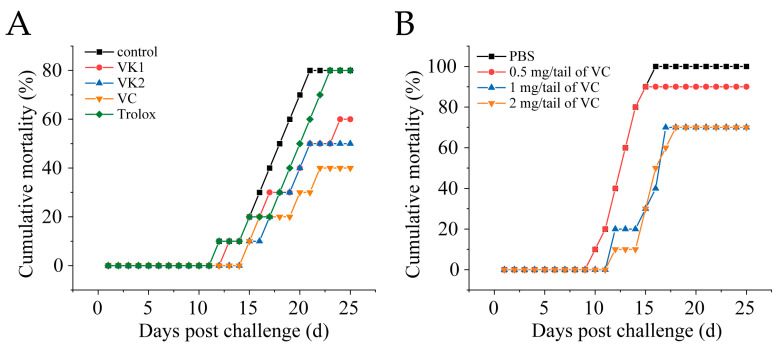
VC reduces mortality in mandarin fish infected with SDDV. (**A**) Mortality rate of SDDV-infected mandarin fish after injection with different antioxidants; (**B**) mortality rate of SDDV-infected mandarin fish after treatment with different concentrations of VC. n = 3.

Mandarin fish were intramuscularly injected with 1 mg/fish of VC, VK1, VK2, or Trolox. After 4 h, the fish were intraperitoneally challenged with SDDV ZH-06/20 (10^3^ TCID_50_/fish). VC, VK1, and VK2 significantly reduced mortality, with VC exhibiting the most pronounced protective effect, lowering the mortality rate from 90% in the control group to 42.5% in the VC group (Figure 2A). To optimize the VC dosage, fish were injected with 0.5 mg/fish, 1 mg/fish, or 2 mg/fish of VC. The results showed 100% cumulative mortality in the control group, while the mortality rates in the 0.5 mg/fish, 1 mg/fish, and 2 mg/fish VC groups were 85%, 65%, and 65%, respectively. Treatment with at least 1 mg/fish of VC provided significantly effective protection against fatal SDDV infection (Figure 2B).

### 3.3. VC Inhibits SDDV Infection in MFF-1 Cells

**Figure 3:**
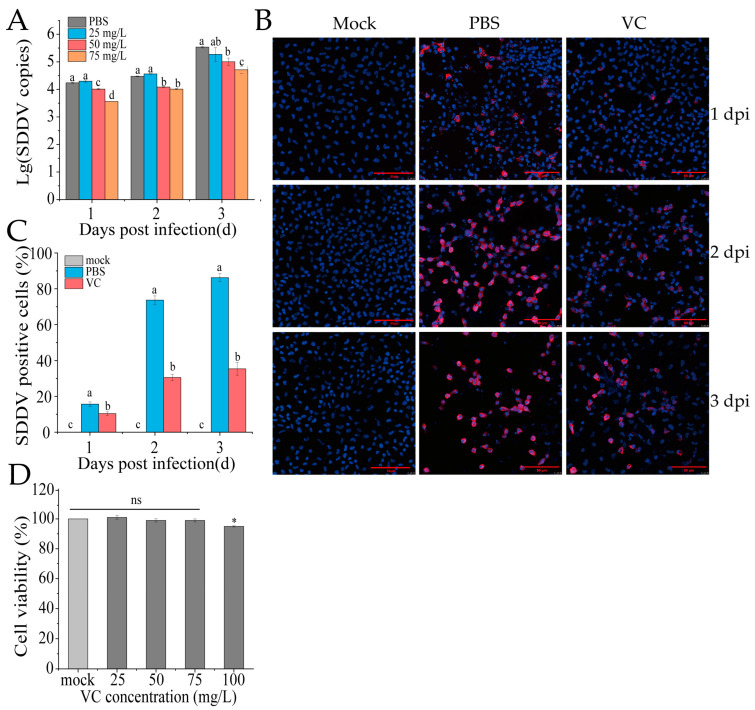
VC inhibits SDDV infection in MFF-1 cells. (**A**) SDDV copies in MFF-1 cells after SDDV infection following incubation with VC at concentrations of 25–75 mg/L; (**B**) IFA results of SDDV infection at 1–3 dpi after VC intervention, the red fluorescence represents SDDV MCP and blue fluorescence indicates the cell nuclei; (**C**) percentage of SDDV MCP-positive cells in MFF-1 cells after SDDV infection; (**D**) effect of VC at concentrations of 25–100 mg/L on cell viability. The data shown are the mean values ± SD of three independent experiments or replicates. A two-tailed unpaired Student’s *t*-test was used for the statistical analysis. ns, not significant; * *p* < 0.05. Different letters (a, b, c, d) indicate significant differences (*p* < 0.05) between groups.

To assess whether VC reduces SDDV infection at the cellular level in vitro, MFF-1 cells were pretreated with VC (25, 50, or 75 mg/mL) at 27 °C for 4 h, followed by SDDV infection (MOI = 5). The PBS group served as the control, where cells were pretreated with PBS instead of VC, and the mock group was treated with PBS without virus infection. Samples were collected at 1, 2, and 3 dpi for DNA extraction and qPCR analysis. The results showed that VC significantly inhibited SDDV replication in a dose-dependent manner compared to the PBS group, with the strongest inhibition effect at 75 mg/mL (Figure 3A). IFA analysis showed markedly reduced SDDV-associated red fluorescence signals at 2 dpi in the VC group relative to the PBS group (Figure 3B). By calculating the proportion of infected cells, we found that the percentage of infected cells in the PBS group was significantly higher than that in the VC group at 1–3 dpi (Figure 3C). Additionally, while an increase in fluorescence intensity in infected cells was visually observed at 3 dpi compared to 2 dpi in the VC group (Figure 3B), the proportion of infected cells did not increase substantially (Figure 3C). This indicates that while VC treatment does not dramatically reduce viral replication, it significantly inhibits the number of infected cells. Moreover, VC showed no cytotoxicity to MFF-1 cells at the tested concentrations (Figure 3D).

### 3.4. VC Inhibits SDDV-Induced Ferroptosis

**Figure 4:**
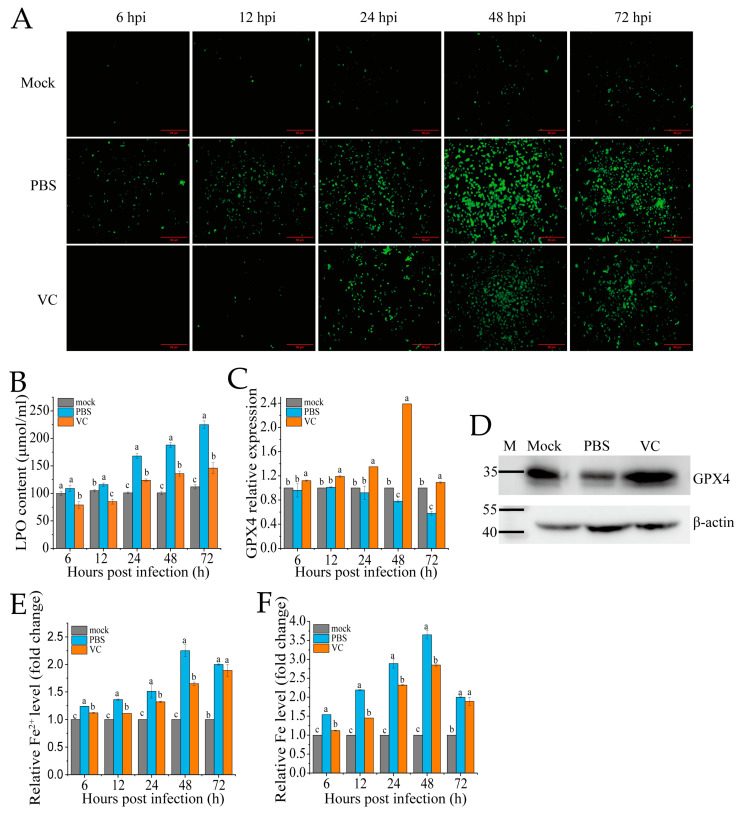
VC suppresses SDDV-induced ferroptosis on MFF-1. Fluorescence of ROS (**A**), LPO (**B**), GPX4 mRNA expression (**C**), and GPX4 protein expression at 48 hpi (**D**); relative Fe^2+^ level (**E**) and relative total iron level (**F**) were measured in the VC-treated and PBS control groups at 6–72 hpi with SDDV. The data shown are the mean values ± SD of three independent experiments or replicates. Statistical analysis was performed using a two-tailed unpaired Student’s *t*-test. Different letters (a, b, c) indicate significant differences (*p* < 0.01) between groups.

To assess whether VC can mitigate SDDV-induced ferroptosis, we pre-incubated the MFF-1 cells with VC prior to infection with SDDV, followed by a series of ferroptosis-related assays in comparison with PBS-treated controls. The accumulation of ROS and LPO are recognized as key events in the initiation of ferroptosis and are hallmark features of ferroptotic cell death [30]. In this study, we employed DCFH-DA and LPO assay kits to evaluate changes in ROS and LPO levels in MFF-1 cells following VC treatment. After SDDV infection, the ROS levels progressively increased between 6 and 72 hpi, as evidenced by increasing green fluorescence signals (Figure 4A). In contrast, the VC group significantly reduced the increase in ROS levels caused by SDDV infection (Figure 4A). LPO analysis revealed that SDDV infection notably increased LPO levels, but VC treatment resulted in a marked reduction in LPO levels compared to the PBS group (Figure 4B). In addition, our findings show that SDDV infection resulted in the downregulation of GPX4 mRNA (Figure 4C) and protein (Figure 4D) levels, whereas VC treatment effectively reversed this downregulation. Ferroptosis, being iron-dependent, is triggered by changes in iron homeostasis [31]. After SDDV infection, both total iron and Fe^2+^ levels were elevated, whereas VC treatment significantly decreased Fe^2+^ and total iron levels compared to the PBS group (Figure 4E,F).

### 3.5. Inhibition of Nrf2 Promotes Ferroptosis and SDDV Infection in MFF-1 Cells

**Figure 5:**
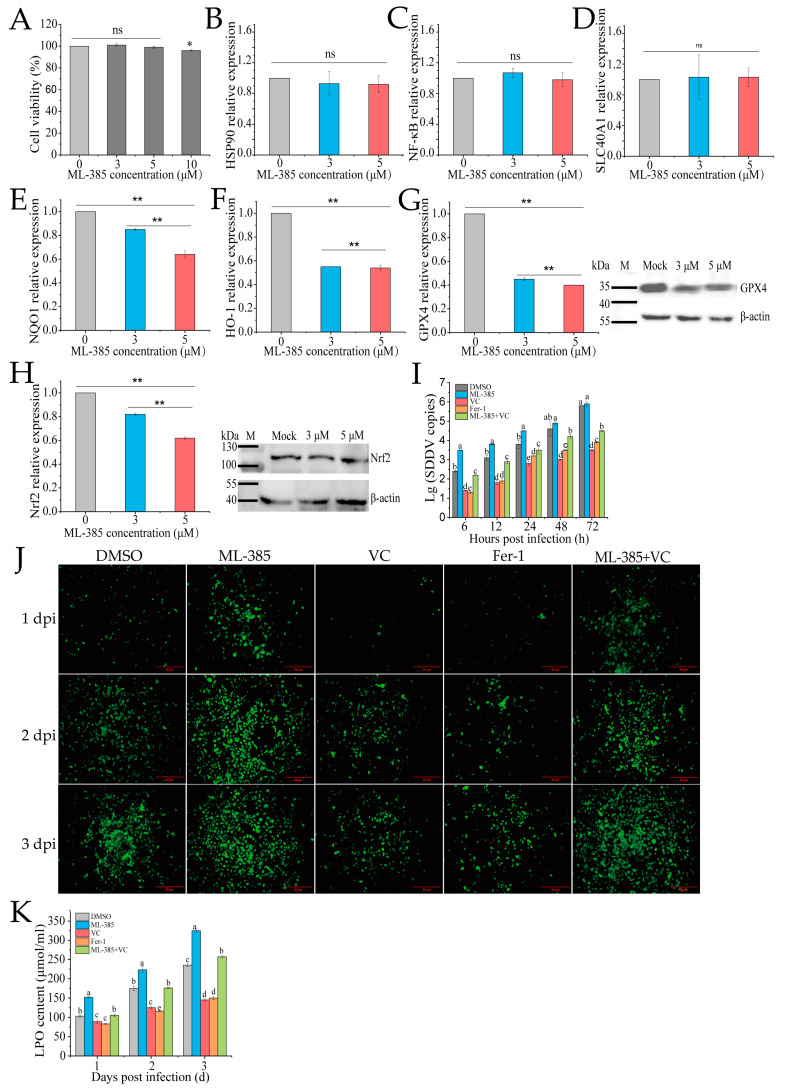
Inhibition of Nrf2 reduces ferroptosis and SDDV replication in MFF-1 cells. (**A**) Treatment with 3 and 5 μM ML-385 did not affect MFF-1 cell viability. After ML-385 incubation, the relative expression levels of *HSP90* (**B**), *NF-kB* (**C**), *SLC40A1* (**D**), *NQO1* (**E**), *HO-1* (**F**), *GPX4* (**G**), and *Nrf2* (**H**) were reduced, with corresponding decreases in the protein expression of both GPX4 and Nrf2 (**G**,**H**). Comparison of SDDV copies (**I**), ROS fluorescence (**J**), and LPO (**K**) levels in MFF-1 cells pretreated with DMSO, ML-385, VC, Fer-1, or ML-385 + VC and subsequently infected with SDDV. The data shown are the mean values ± SD of three independent experiments or replicates. Statistical analysis was performed using a two-tailed unpaired Student’s *t*-test. ns, not significant; * *p* < 0.05; ** *p* < 0.01. Different letters (a, b, c, d, e) indicate significant differences between groups.

Nrf2 regulates oxidative stress and ferroptosis by modulating antioxidant enzymes and iron metabolism genes. To explore the role of Nrf2 in VC-mediated inhibition of SDDV-induced ferroptosis in MFF-1 cells, we used the Nrf2 inhibitor ML385. ML385 binds to the Neh1 domain of Nrf2, preventing the Nrf2-MAFG complex from interacting with the ARE sequence, thus inhibiting the expression of downstream target genes [32]. CCK-8 assays showed that 3 µM and 5 µM ML385 did not affect cell viability (Figure 5A). After ML385 treatment, qRT-PCR confirmed that ML-385 did not affect the expression of Nrf2-unrelated genes, including *HSP90*, *NF-κB*, and *SLC40A1* (Figure 5B–D), while it significantly downregulated the mRNA expression of *Nrf2* and its downstream targets, including *HO-1*, *NQO1*, and *GPX4* (Figure 5E–H), demonstrating that ML-385 specifically inhibits the *Nrf2* pathway. Western blotting further confirmed a reduction in the protein levels of both GPX4 and Nrf2, with the most significant decrease observed at 5 µM (Figure 5G,H). Therefore, 5 µM was used in further experiments. The groups were as follows: DMSO (ZH-06/20, MOI = 5), ML-385 (5 µM + ZH-06/20, MOI = 5), VC (75 mg/mL VC + ZH-06/20, MOI = 5), positive control (10 µM ferrostatin-1 + ZH-06/20, MOI = 5), and ML-385 + VC (5 µM ML385 + 75 mg/mL VC + ZH-06/20, MOI = 5). The qPCR results showed that ferrostatin-1 and VC treatment significantly reduced SDDV copy numbers, while the ML-385 + VC combination reversed VC’s inhibitory effect (Figure 5I).

Further investigation of ROS and LPO levels showed that ML-385 reversed VC’s inhibitory effect on these markers. The ML-385 + VC combination group exhibited significantly higher ROS and LPO levels compared to the VC treatment alone (*p* < 0.01) (Figure 5J,K). This suggests that Nrf2 plays a crucial role in VC’s inhibition of ferroptosis. Inhibiting Nrf2 decreased antioxidant gene expression, weakening antioxidant defense and increasing ROS and LPO.

### 3.6. VC Inhibits SDDV-Induced Ferroptosis by Activating Nrf2 Nuclear Translocation

**Figure 6:**
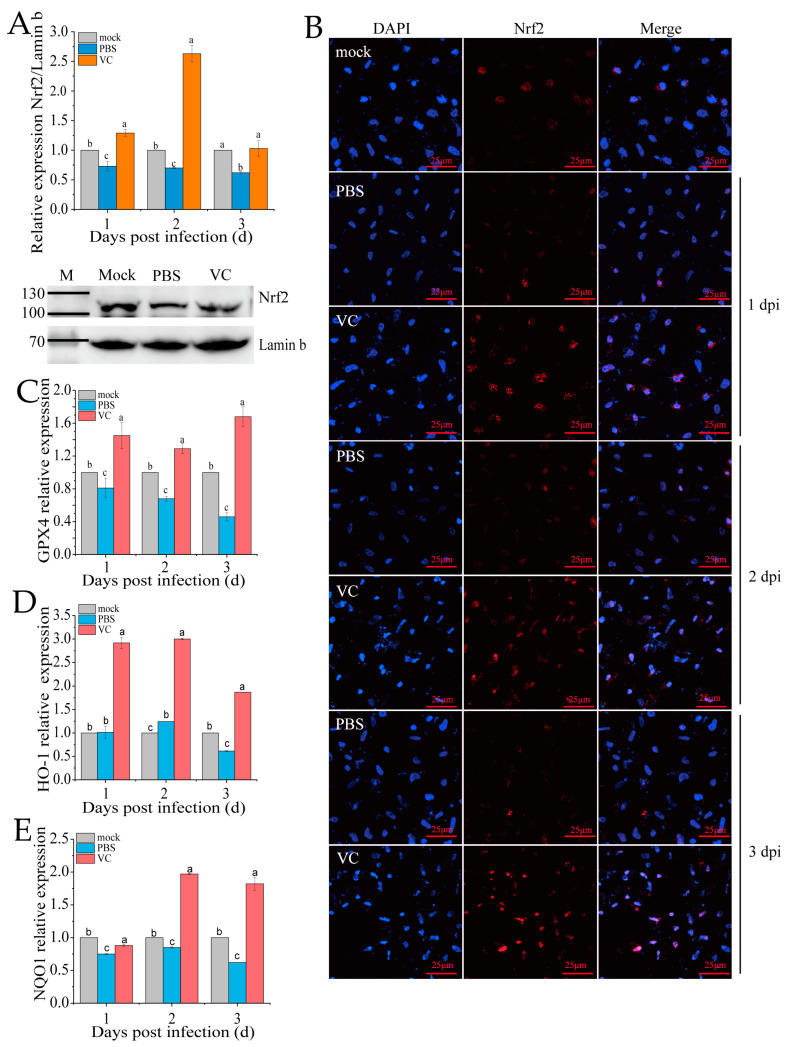
Effect of VC on Nrf2 distribution in SDDV-induced ferroptosis in MFF-1 cells. (**A**) Nrf2 mRNA expression in the nucleus and protein expression at 2 dpi were measured in MFF-1 cells infected with SDDV at 1–3 dpi in the VC and PBS control groups; (**B**) fluorescence imaging shows the expression and localization of Nrf2 protein following SDDV infection in the VC and PBS control groups; (**C**–**E**) the relative expression levels of *GPX4*, *HO-1*, and *NQO1* in MFF-1 cells infected with SDDV at 1–3 dpi in the VC and PBS control groups were also compared. The data shown are the mean values ± SD of three independent experiments or replicates. Statistical analysis was performed using a two-tailed unpaired Student’s *t*-test. Different letters (a, b, c) indicate significant differences between groups.

Under typical physiological states, Nrf2 is sequestered in the cytoplasm through Keap1-mediated retention, followed by proteasomal degradation to ensure minimal intracellular concentrations [11]. The nuclear translocation of Nrf2 is a key marker of its activation in the Nrf2-ARE signaling pathway. Following VC treatment and subsequent SDDV infection, qRT-PCR analysis revealed a significant upregulation of *Nrf2* expression in the nucleus compared to the PBS group (Figure 6A). Additionally, nuclear Nrf2 protein levels were markedly higher in the VC group than in the PBS control (Figure 6A). To further assess Nrf2 distribution, we conducted immunofluorescence staining in the PBS and VC groups (Figure 6B). The results showed that the red fluorescence representing Nrf2 in the PBS group was markedly weaker, indicating a significant decrease in Nrf2 levels following SDDV infection. In the VC group, the red fluorescence was significantly enhanced, suggesting that VC treatment activated Nrf2 expression. Moreover, co-localization analysis of Nrf2 with the cell nucleus revealed a clear reduction in nuclear Nrf2 content in the PBS group. In contrast, in the VC group, the red fluorescence was significantly enhanced and translocated into the nucleus, which in turn stimulated the expression of downstream Nrf2 target genes (*GPX4*, *HO-1*, and *NQO1*) (Figure 6C–E). These findings suggest that VC not only promotes Nrf2 expression but also induces its translocation to the nucleus, thereby contributing to cellular protection.

### 3.7. VC Suppresses Inflammation Induced by SDDV in Mandarin Fish

**Figure 7:**
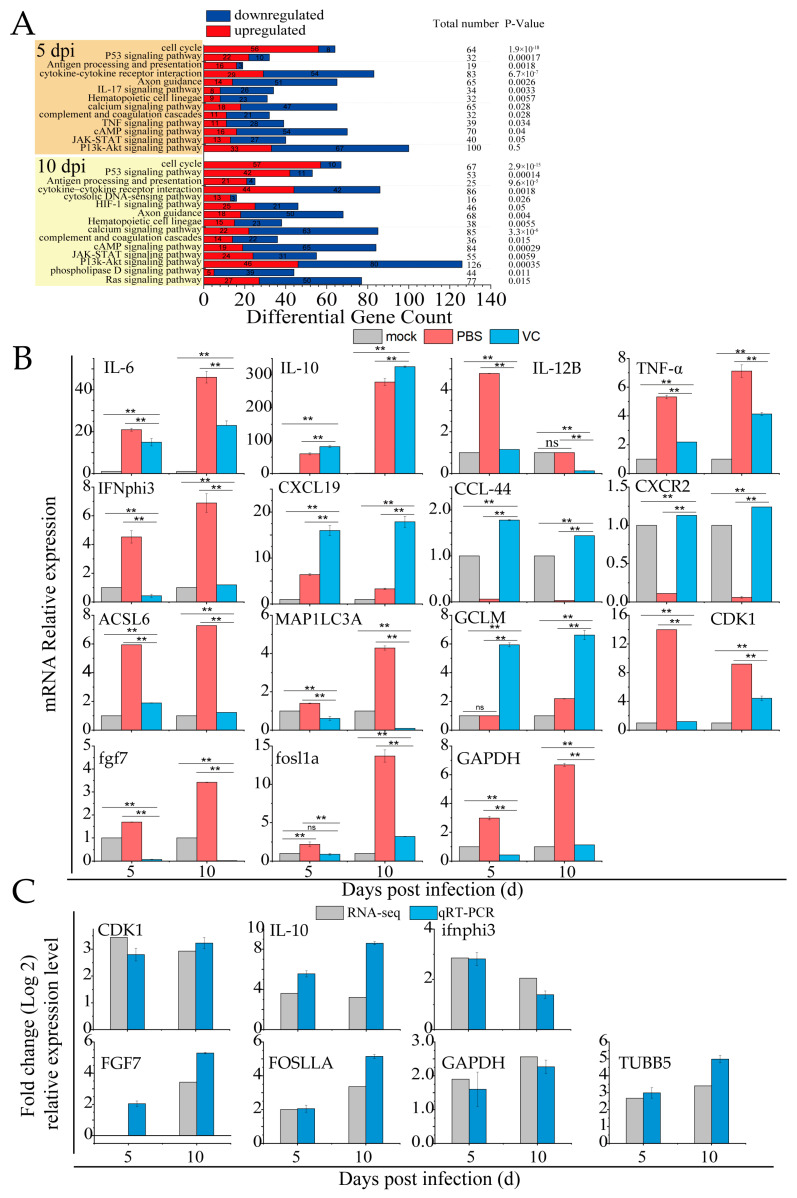
VC suppresses the inflammation induced by SDDV in mandarin fish. (**A**) Enrichment of innate immune-related signaling pathways induced by SDDV infection (*p* < 0.05); (**B**) relative expression levels of *IL-6*, *IL-10*, *IL-12B*, *TNFα*, *IFNphi3*, *CXCL19*, *ACSL6*, *MAP1LC3C*, *GCLM*, *CDK1*, *fgf7*, *fosl1a*, *GAPDH*, *CCL44*, and *CXCR2* in the VC and PBS control groups after SDDV infection; (**C**) qRT-PCR validation of differentially expressed genes identified in the RNA-seq analysis. The data shown are the mean values ± SD of three independent experiments or replicates. Statistical analysis was performed using a two-tailed unpaired Student’s *t*-test. ns, not significant; ** *p* < 0.01.

RNA-seq analysis of spleen tissues from mandarin fish infected with SDDV identified key immune-related biological functions, particularly pathways involved in the cell cycle, P53-mediated tumor suppression, antigen processing/presentation, the complement/coagulation cascades, and cytokine–cytokine receptor interactions, and the IL-17, JAK-STAT, and PI3K-Akt signaling pathways (Figure 7A). These pathways are crucial for immune and inflammatory regulation. SDDV infection activates the innate immune response, notably through changes in the IL-17 and cytokine pathways, suggesting that inflammation may become over-activated, potentially leading to tissue damage or immune pathology. Alterations in the complement and coagulation pathways indicate possible disruptions in coagulation processes, while JAK-STAT signaling changes may reflect immune cell dysfunction. In summary, SDDV infection triggers a robust immune and inflammatory response.

Table 2 highlights the differentially expressed genes in innate immune signaling pathways during SDDV infection. In the PBS group, the qRT-PCR results showed significant upregulation of inflammatory genes such as *IL-6*, *IL-10*, *IL-12B*, *TNFα*, *IFNphi3*, *CXCL19*, *ACSL6*, *MAP1LC3C*, *GCLM*, *CDK1*, *fgf7*, *fosl1a*, and *GAPDH*, alongside a downregulation of *CCL44* and *CXCR2* (Figure 7B). These findings, which are consistent with the transcriptomic data, confirm that SDDV infection triggers immune and inflammatory responses. In contrast, the VC group exhibited significant downregulation of *IL-6*, *IL-12B*, *TNFα*, *IFNphi3*, *ACSL6*, *MAP1LC3C*, *CDK1*, *fgf7*, *fosl1a*, and *GAPDH*, with upregulation of *IL-10*, *CXCL19*, *GCLM*, *CCL44*, and *CXCR2* compared to the PBS group. This suggests that VC injection modulates immune and inflammatory responses, likely by reducing inflammation or altering immune cell signaling.

## 4. Discussion

Our preliminary studies showed that SDDV infection in mandarin fish results in a pronounced ferroptosis phenomenon [9]. By applying various inhibitors of different cell death pathways and ferroptosis inducers to MFF-1 cells, we demonstrated that ferroptosis is the predominant mode of cell death triggered by SDDV [9]. The peroxidation of phospholipids and the accumulation of lipid ROS are the central drivers of ferroptosis-mediated cell death. Notably, numerous studies have reported significant success in inhibiting ferroptosis by using antioxidants to reduce ROS levels [33,34,35]. Therefore, targeting ferroptosis through antioxidant intervention represents a potentially effective therapeutic strategy for SDDV infection.

The RNA-seq analysis of spleens from SDDV-infected mandarin fish identified 650 DEGs related to cell death (Figure 1B). Consistent with our previous findings, KEGG pathway analysis revealed that SDDV primarily induces spleen cell damage through ferroptosis and apoptosis (Figure 1B), with ferroptosis playing a dominant role. Pathway analysis indicated that the P53, IL-17, and PI3K-Akt pathways are key regulators of cell death (Figure 1C). P53 activation promotes apoptosis and may exacerbate ferroptosis by inducing oxidative stress [36,37]. The IL-17 pathway enhances ROS generation, potentially amplifying ferroptosis [38]. The activation of the PI3K pathway leads to the production of various growth factors and subsequently activates the downstream protein Akt, which is involved in regulating cell proliferation and differentiation [39]. Yi et al. (2020) reported that activation of the PI3K-AKT-mTOR signaling pathway can inhibit ferroptosis [40]. Additionally, research has demonstrated that the PI3K/AKT pathway facilitates the phosphorylation of FOXO1, resulting in its translocation from the nucleus to the cytoplasm and a consequent loss of transcriptional activity [41]. These pathways interact to regulate SDDV-induced cell death. Additionally, ferroptosis-related DEGs associated with iron metabolism, oxidative stress, and lipid metabolism were identified (Figure 1D). SDDV infection upregulated genes such as *TF*, *STEAP3*, and *NCOA4*, enhancing iron uptake but downregulating *TFRC* and *SLC40A1*, leading to iron overload. Increased oxidative stress was indicated by the downregulation of *GPX4* and upregulation of *GCLM*, promoting ROS accumulation and lipid peroxidation. The results indicate that SDDV infection alters the iron and lipid metabolic processes, thereby increasing susceptibility to ferroptosis and highlighting potential therapeutic targets for intervention.

In this study, we conducted antioxidant treatment trials in SDDV-infected mandarin fish, aiming to identify the most effective agent in mitigating the infection-induced cellular damage. Our results demonstrated that VC exhibited the strongest inhibitory effect (Figure 2A). Further in vitro validation confirmed that VC significantly reduced SDDV replication (Figure 3A). However, at 1–3 dpi, the viral load was only reduced by a maximum of 1 log, which was insufficient to support VC as a strong inhibitor of SDDV infection. In contrast, IFA analysis showed that VC significantly reduced the number of infected cells compared to the PBS group (Figure 3C). Although fluorescence intensity increased at 2–3 dpi (Figure 3B), the increase in the proportion of infected cells was minimal, suggesting that VC may reduce cellular damage and infection by inhibiting ferroptosis and enhancing immune defense mechanisms.

VC functions as a powerful antioxidant through two distinct mechanisms: it directly interacts with aqueous peroxyl radicals and indirectly enhances the antioxidant capabilities of fat-soluble vitamin E [42]. These actions collectively mitigate LPO- and ROS-induced oxidative damage in cellular membranes, both at the cell surface and within organelles, while also protecting non-lipid nuclear materials. However, despite its well-known antioxidant properties, the role of VC in ferroptosis inhibition remains underexplored [17]. Some studies suggest that VC may exert protective effects against ferroptosis, with its function as either a pro-oxidant or antioxidant depending on its concentration. At low concentrations (μM), VC reduces ROS levels, whereas at higher concentrations (mM), it can induce ROS production [43]. In our study, treatment with VC (25–50 mg/L) effectively decreased SDDV-induced increases in LPO, ROS, and iron content while enhancing GPX4 levels, thereby inhibiting SDDV-induced ferroptosis (Figure 4).

Nrf2, a bZIP transcription factor, is a central regulator of cellular antioxidant defenses and counteracts oxidative stress by orchestrating endogenous protective mechanisms. Its functionality is regulated by Keap1, which sequesters Nrf2 in the cytoplasm and directs it towards proteasomal degradation [44,45]. Nrf2 regulates several key factors involved in ferroptosis, including GPX4 protein levels [11], the free intracellular iron content [46], mitochondrial health [47], and NADPH regeneration [48], thereby influencing ferroptosis both directly and indirectly. Research has demonstrated that antioxidants can inhibit ferroptosis via the Nrf2 signaling pathway. For instance, Ma et al. (2020) illustrated that melatonin mitigates high-glucose-induced ferroptosis in type 2 diabetic mice by activating the Nrf2/HO-1 pathway [49]. Similarly, Ouyang et al. found that Angelica polysaccharides suppress ferroptosis by activating Nrf2 [50]. ML-385, an Nrf2 inhibitor that has been confirmed to specifically downregulate Nrf2 and its downstream antioxidant factors [32], is widely used in research involving the Nrf2 pathway [51,52,53,54]. In our study, we treated MFF-1 cells with ML-385 and VC prior to SDDV infection. We found that ML-385 counteracted VC’s inhibition of ferroptosis, as shown by increased LPO and ROS levels (Figure 5J,K), and prevented VC from inhibiting SDDV replication in MFF-1 cells (Figure 5I), suggesting that VC’s effect on ferroptosis is Nrf2-dependent. Further analysis revealed that SDDV infection decreased *Nrf2* expression, likely due to the activation of the cellular defense system. In the VC group, Nrf2 expression significantly increased, with enhanced nuclear accumulation (Figure 6A,B). IFA confirmed that VC treatment elevated Nrf2 levels and facilitated its nuclear translocation (Figure 6C), activating the Nrf2 pathway and leading to the activation of downstream antioxidant genes such as *HO-1*, *GPX4*, and *NQO1* (Figure 6D). These results support VC’s role in alleviating ferroptosis through Nrf2 activation and highlight the potential of antioxidants as therapeutic strategies against ferroptosis.

Immune-related signaling pathways were primarily enriched in the P53, IL-17, TNF, JAK-STAT, PI3K-Akt, and HIF-1 signaling pathway after SDDV infection, as revealed by the RNA-seq analysis of mandarin fish spleens (Figure 7A). This suggests that SDDV infection induces a strong inflammatory response in the host. After SDDV infection, the upregulation of *IL-6*, *IL-12B*, *TNFα*, *IFNphi1*, *IFNphi3*, and *CXCL19* indicated a strong immune and inflammatory response (Figure 7C). *IL-6*, *TNFα*, and *IL-12B* activate the NF-κB and JAK-STAT signaling pathways, promoting immune cell recruitment and inflammatory cytokine secretion, thereby exacerbating local inflammation. *CXCL19*, as a chemokine, activates the MAPK pathway through its receptor *CXCR3*, further promoting immune cell migration to the infection site. Additionally, the upregulation of *ACSL6* and *MAP1LC3C* is linked to lipid metabolism and autophagy, and these genes also modulate NF-κB and PI3K-Akt pathways, amplifying immune responses and inflammation, which further contribute to cellular damage. VC has been shown to mitigate inflammation through various mechanisms, including the inhibition of ROS production, modulation of inflammatory mediators, reduction of immune cell infiltration, prevention of endothelial dysfunction, enhancement of microcirculation, and alleviation of a micro-inflammatory state [22,23,42,43]. In the VC group, the downregulation of *IL-6*, *IL-12B*, *TNFα*, *IFNphi1*, *IFNphi3*, and other inflammation-related genes suggests that VC effectively suppressed excessive inflammation. By activating the Nrf2 pathway and regulating the expression of *GCLM*, VC enhanced antioxidant responses, with Nrf2 controlling antioxidant enzymes and glutathione synthesis to reduce oxidative damage. Additionally, the upregulation of *IL-10* indicates that VC activated anti-inflammatory pathways, inhibiting the overactivation of immune responses. The upregulation of *CCL44* and *CXCR2* further suggests that VC, through Nrf2 activation, enhanced both anti-inflammatory and immune responses. Through the modulation of these factors, VC suppressed the immune and inflammatory responses induced by SDDV, thereby alleviating the cellular damage caused by the infection.

## 5. Conclusions

Our study demonstrated that VC effectively inhibits the ferroptosis induced by SDDV infection by activating the Nrf2 pathway, thereby alleviating cellular damage. SDDV infection triggers a robust immune and inflammatory response, promoting ROS generation and exacerbating ferroptosis. In vivo, VC reduced the mortality rate of fish infected with SDDV. In vitro, VC inhibited SDDV infection by reducing the infection rate compared to the control group. By activating the Nrf2 pathway, VC regulates the expression of key antioxidant enzymes to reduce oxidative damage, thereby inhibiting the ferroptosis induced by SDDV. Additionally, VC lowers the levels of inflammatory factors and upregulates anti-inflammatory factors, further enhancing its anti-inflammatory effects. Overall, VC offers a novel therapeutic approach for combating ferroptosis in SDDV infection.

## Figures and Tables

**Table 1:** Primers used in this study.

Gene	GenBank Accession No.	Primer	Primer Sequence (5′-3′)
*β-actin*	AY885683	β-actin-F	AGAGGGAAATCGTGCGTG
		β-actin-R	GAAGGAAGGCTGGAAGAGG
*Nrf2*	MT270449	Nrf2-F	AGACGAAAGCGAAAGCTCCT
		Nrf2-R	GCTCTCTTCCAGAATGGCGT
*GPX4*	XM_044200772	GPX4-F	ACGCATCCTTGCTTTCCCTT
		GPX4-R	TGCTCTTTCAGCCACTTCCA
*HO-1*	KJ765334	HO-1-F	CCAGAAGGGACAGATCACCC
		HO-1-R	CCTCCTCTAGCGCCTTGTAG
*NQO1*	XM_044189953	NQO1-F	CCAGCATGGGTGACCTGAA
		NQO1-R	CTCTGTCCATCCAGCCCTTC
*IL-6*	MT126997	IL-6-F	AGCCCATCGCAGAGAAAAGG
		IL-6-R	GCACTTGTTGAGTTTCCGCT
*IL-10*	MH644036	IL-10-F	TTATCTGGGCACGGTTCTGC
		IL-10-R	GGACTCCATGTGCGGCTTTA
*IL-12B*	XM_044195110	IL-12B-F	AGGATGCCAACTGCCCATAC
		IL-12B-R	TGAACACCGTCCCATCACTG
*TNFα*	XM_044171435	TNFα-F	TCGTATGGGAACGATGACGC
		TNFα-R	ATCGCTGCTGGCTGTCTTTT
*IFNphi3*	XM_044181379	IFNphi3-F	TCACCTGGGACAATGCGAAA
		IFNphi3-R	TGGACAAACAGCTTCCCTCC
*CXCL19*	XM_044184584	CXCL19-F	TGCTTGAACCCTCGGTCTTC
		CXCL19-R	CTTGGGAAGTGCTTGTCCCT
*CCL44*	XM_044200386	CCL44-F	TGCTGCATGTTGTACTCCCA
		CCL44-R	GTCTCTGGGGTCTGCACATT
*CXCR2*	XM_044217766	CXCR2-F	AATGTGCAGACCCCAGAGAC
		CXCR2-R	AGGAAGGTGCCACATGGTTC
*ACSL6*	XM_044221798	ACSL6-F	CTATCAGGGCGACATCCGTC
		ACSL6-R	CAGCCAACGCTTCAATGGAC
*MAP1LC3A*	XM_044183152	MAP1LC3A-F	AGGAGGACATTTGCTGACCG
		MAP1LC3A-R	ATCACGGGGATCTTGTTGGG
*GCLM*	XM_044199155	GCLM-F	TGAAGCCCAGCAGTAACCAG
		GCLM-R	CCAGTCAGCGATGTTCAGGT
*CDK1*	XM_044213061	CDK1-F	GAGTTCTTCACCGGGACCTG
		CDK1-R	CGACAGGGGTTGAGTATCGG
*fgf7*	XM_044189235	fgf7-F	CCTCCACAACTACCCTGTCG
		fgf7-R	ACCCTTGTGTTGTGACCTCC
*fosl1a*	XM_044204836	fosl1a-F	GGCTCACCGTCCCATTTGTA
		fosl1a-R	GCTTGCAGTTTGGTTGAGGG
*GAPDH*	XM_044209071	GAPDH-F	CCAGAACATCATCCCCGCTT
		GAPDH-R	CGTCGTATTTGGCGGGTTTC
*tubb5*	XM_044209030	tubb5-F	ACAGGGACTTACCACGGAGA
		tubb5-R	AAGGGTCCAGACCTCACAGA
*FGF7*	XM_044189235	FGF7-F	CCTCCACAACTACCCTGTCG
		FGF7-R	ACCCTTGTGTTGTGACCTCC
*MAP1LC3C*	XM_044189420	MAP1LC3C-F	TACGAGGAAACACGAGGTGG
		MAP1LC3C-R	ACTTGGTTTTGTCCAGCAGC
*NF-κB*	EF492047	NF-κB-F	ACAACAGGACAGACTGGCTC
		NF-κB-R	CTGGATTGCCAGAAGGACCC
*HSP90*	XM_044180633	HSP90-F	TAGCCCACGCTAGGTTCTTG
		HSP90-R	TTCGGGTGGGTGTCATCTTC
*SLC40A1*	XM_044217457	SLC40A1-F	TGATCCGCACAGGCTTCATT
		SLC40A1-R	CAAAGACAGAGGCAACGCAC

**Table 2:** Highly expressed differential genes in immune system pathways induced by SDDV infection.

KEGG Pathway	Pathway ID	Differentially Expressed Genes
Cell cycle	hsa04110	*LOC122879039, BUB1, BUB1BB, CCNA2, CCNB1, CCNB3, CDC20, CDK1, CDKN2C, DBF4B, ESPL1, MAD2L1, PLK1, CCNDX, CDK18*
p53 signaling pathway	hsa04115	*LCCNB1, CDC20, CDK1, CTSL.1, IGFBP3, SERPINE1, TUBB5, TUBB6, CCNDX, CDK18*
Antigen processing and presentation	hsa04612	*CTSL.1, CTSLA, HSPA1B, PSME2*
Cytokine–cytokine receptor interaction	hsa04060	*BMP7B, CXCL19, IFNphi3, IL10, IL12BB, NRADD, VEGFAA, LOC122871787, CCL44, CXCL12B, CXCR2, PRLRA*
Cytosolic DNA-sensing pathway	hsa04623	*LOC122864465, CGASA, CXCL19, IFNphi3*
HIF-1 signaling pathway	hsa04066	*ANGPT2A, ANGPTL4, CPB2, EGLN2, EGLN3, ENO1A, GAPDHS, SERPINE1, SLC2A3B, VEGFAA, LOC122885592, CPB1, EPAS1B, GAPDH, PFKFB1*
Axon guidance	hsa04360	*CPB2, LINGO2B, ABLIM1A, ABLIM3, CPB1, CXCL12B, EPHB6, LRRC4BA, NTNG1A, SEMA3AB*
IL-17 signaling pathway	hsa04657	*FOSL1A, CCL44, ELAVL4, FOSAB, FOSB, MMP9*
Hematopoietic cell lineage	hsa04640	*LOC122864664, LOC122879019, ITGA8*
Calcium signaling pathway	hsa04020	*CHRNA11, CPB2, AGTR1B, ATP2B2, AVPR1AA, CACNA1EB, CACNA1IA, CCKAR, CPB1, DRD1B, GRIN2DA, GRM6B, HTR2CL1, NOS1, OXTRA P2RX2 P2RX4B PLCD1B STIM2A TACR2 TRHRA TRHRB*
Complement and coagulation cascades	hsa04610	*C8GF2, FGF13B, SERPINF2B, SSTR1A*
TNF signaling pathway	hsa04668	*LOC122864664, MMP19, LOC122876711, LOC122888378, FOSAB, FOSB, MMP9*
cAMP signaling pathway	hsa04024	*CNGA1B, CPB2, JUNE, AGTR1B, ATP2B2, DRD1B, DRD2L, FOSAB, FOSB, GRIN2DA, SSTR1A, VIPR2*
JAK-STAT signaling pathway	hsa04630	*IFNPHI3, IL10, IL13RA2*
PI3K-Akt signaling pathway	hsa04151	*FGF7, IFNPHI3, MYBL1, PDGFC, CCNDX, PRLRA*
Phospholipase D signaling pathway	hsa04072	*AGTR1B, CPB1, CXCR2, DNM3B, GRM3, GRM6B, OXTRA, SHC2*
Ras signaling pathway	hsa04014	*FGF7, PDGFC, FGFR1B, FGFR4, GNB3B, GRIN2DA, LRRC74B, NTRK2A*

## Data Availability

Datasets used or analyzed in this study are available from the corresponding author upon reasonable request.

## Abbreviations

The following abbreviations are used in this manuscript:

SDDV	Scale drop disease virus
VC	Vitamin C
DEGs	Differentially expressed genes
ROS	Reactive oxygen species
LPO	Lipid peroxidation
Nrf2	Nuclear factor erythroid 2-related factor 2
DMEM	Dulbecco’s modified Eagle’s medium
FBS	Fetal bovine serum
MCP	Major capsid protein
SD	Standard deviation
ANOVA	Analysis of Variance
